# Supplementary material for: Inhibiting IRE1α-endonuclease activity decreases tumor burden in a mouse model for hepatocellular carcinoma
Source: eLife. 2020 Oct 26;9:e55865. doi: 10.7554/eLife.55865 (PMC7661042; doi:10.7554/eLife.55865)
Supplement: Supplementary file 1. [file elife-55865-supp1.docx]

| **Gene Name** | **NCBI Reference** | **Species** | **Primer** | **Comments** |
| --- | --- | --- | --- | --- |
| *ACTA2* | NM_001613.3 | Human | F: GACAGCTACGTGGGTGACGAA R: TTTTCCATGTCGTCCCAGTTG | Stellate cell activation |
| *HSPA5* | NM_005347.4 | Human | F: GAAAGAAGGTTACCCATGCAGT R: CAGGCCATAAGCAATAGCAGC | ER-stress |
| *Hspa5* | NM_001163434.1 | Mouse | F: GTGTGTGAGACCAGAACCGT R: TAGGTGGTCCCCAAGTCGAT | ER-stress |
| *DDIT3* | NM_001195057.1 | Human | F: CATCACCACACCTGAAAGCA R: TCAGCTGCCATCTCTGCA | ER-stress |
| Collagen Iɑ2 | NM_000089.3 | Human | F: GGCCCTCAAGGTTTCCAAGG R: CACCCTGTGGTCCAACAACTC | Stellate cell activation |
| *GAPDH* | NM_001289745.2 | Human | F: GGAGCGAGATCCCTCCAAAAT R: GGCTGTTGTCATACTTCTCATGG | Reference Gene |
| *Gapdh* | XM_017321385.1 | Mouse | F: AATGAAGGGGTCGTTGATG R: GGTGAAGGTCGGTGTGAAC | Reference Gene |
| *ERN1* | NM_001433.4 | Human | F: TAGTCAGTTCTGCGTCCGCT R: TTCCAAAAATCCCGAGGCCG | ER-stress |
| *MMP1* | NM_001145938.1 | Human | R: GGTCTCTGAGGGTCAAGCAG F: AGTTCATGAGCTGCAACACG | Metastasis |
| *MMP9* | NM_004994.2 | Human | F: TTGACAGCGACAAGAAGTGG R: GCCATTCACGTCGTCCTTAT | Metastasis |
| *PCNA* | NM_002592.2 | Human | F: AGGCACTCAAGGACCTCATCA R: GAGTCCATGCTCTGCAGGTTT | Proliferation |
| *Pcna* | NM_011045.2 | Mouse | F: AGGCACTCAAGGACCTCATCA R: GAGTCCATGCTCTGCAGGTTT | Proliferation |
| *TBP1* | NM_003194.4 | Human | F: AGTGACCCAGCATCACTGTTT R: GGCAAACCAGAAACCCTTGC | Reference Gene |
| *XBP1* - Spliced | NM_001079539.1 | Human | F: AGACAGCGCTTGGGGATGGAT R: CCTGCACCTGCTGCGGACTC | ER-stress |
| *XBP1* - Unspliced | NM_005080.3 | Human | F: AGACAGCGCTTGGGGATGGAT R: CCTGCTGCAGAGGTGCACGTAG | ER-stress |
| β-Actin | NM_007393.5 | Mouse | F: AAGAGCTATGAGCTGCCTGA R: TACGGATGTCAACGTCACAC | Reference Gene |
| β-ACTIN | NM_001101.4 | Human | F: TCTACAATGAGCTGCGTGTG R: AGCCTGGATAGCAACGTACA | Reference Gene |
| *ATF6* | NM_007348.4 | Human | F: TCAGACAGTACCAACGCTTATGC  R: GTTGTACCACAGTAGGCTGAGA | ER-stress |
| *ATF4* | XM_017028807.2 | Human | F: TGGCCAAGCACTTCAAACCT  R: GTTGTTGGAGGGACTGACCAA | ER-stress |
| *Atf4* | NM_009716.3 | Mouse | F: CTACTAGGTACCGCCAGAAG  R: GCCTTACGGACCTCCTCTAT | ER-stress |
| *EIF2AK3* | NM_001313915.1 | Human | F: ACGATGAGACAGAGTTGCGA  R: GCGCGTAAACAAGTTGCCT | ER-stress |
| *Eif2ak2* | XM_011241202.3 | Mouse | F: GTGACTGCGGACAAAGCAGA  R: CTAAGCCGGGTGTTGCATTC | ER-stress |
| *GADD34* | NM_014330.3 | Human | F: GCTGTCACGCAATCCCTTGT  R: CCAGACAGCCAGGAAATGGA | ER-stress |
| *EDEM1* | XM_011534271.3 | Human | F: CAAGTGTGGGTACGCCACG  R: AAAGAAGCTCTCCATCCGGTC | ER-stress |
| *Edem1* | NM_138677.2 | Mouse | F: CTACCTGCGAAGAGGCCG  R: GTTCATGAGCTGCCCACTGA | ER-stress |
| *HERP* | NM_001010989.3 | Human | F: CATTTAGACCGAGGCCGGTT  R: AGAGCCTGGCATATCACGTC | ER-stress |
| *Herp* | NM_001344134.1 | Mouse | F: ATGGTGGTCCTCGAGATGCT  R: TTTACAAGTCTGCAAGCTCAGG | ER-stress |
| *Ero1b* | NM_026184.2 | Mouse | F: CACTCGGCAGGAAATCGTTG  R:AGCAAACCAAACCAAGGCAT | ER-stress |
| *Grp94* | NM_011631.1 | Mouse | F: AAGAATGAAGGAAAAACAGGACAAAA  R: CAAATGGAGAAGATTCCGCC | ER-stress |
