# Supplementary material for: Inhibiting IRE1α-endonuclease activity decreases tumor burden in a mouse model for hepatocellular carcinoma
Source: eLife. 2020 Oct 26;9:e55865. doi: 10.7554/eLife.55865 (PMC7661042; doi:10.7554/eLife.55865)
Supplement: Supplementary file 2. [file elife-55865-supp2.docx]

| **Target** | **Species** | **Company** | **Product number** |
| --- | --- | --- | --- |
| spliced XBP1 | Goat | Abcam | Ab85546 |
| Total XBP1 | Rabbit | Abcam | Ab37152 |
| IRE1a | Rabbit | Abcam | Ab37073 |
| p-IRE1 | Rabbit | AbNova | PAB12435 |
| aSMA | Rabbit | ThermoFisher | 710487 |
| aSMA | Goat | Abcam | Ab21027 |
| BIP | Rabbit | Abcam | ab21685 |
